# High flow nasal therapy versus noninvasive ventilation as initial ventilatory strategy in COPD exacerbation: a multicenter non-inferiority randomized trial

**DOI:** 10.1186/s13054-020-03409-0

**Published:** 2020-12-14

**Authors:** Andrea Cortegiani, Federico Longhini, Fabiana Madotto, Paolo Groff, Raffaele Scala, Claudia Crimi, Annalisa Carlucci, Andrea Bruni, Eugenio Garofalo, Santi Maurizio Raineri, Roberto Tonelli, Vittoria Comellini, Enrico Lupia, Luigi Vetrugno, Enrico Clini, Antonino Giarratano, Stefano Nava, Paolo Navalesi, Cesare Gregoretti, the H. F.-AECOPD study investigators, Lorenzo Ball, Tiziana Bove, Raffaele Campisi, Paola Chirco, Maria Stella Dionisi, Mariachiara Ippolito, Riccardo Fantini, Luca Guidelli, Uberto Maccari, Luca Tabbì, Maria Rita Taliani

**Affiliations:** 1 Department of Surgical, Oncological and Oral Science (Di.Chir.On.S.), University of Palermo, Palermo, Italy; 2 Department of Anesthesia, Intensive Care and Emergency, Policlinico Paolo Giaccone, University of Palermo, Palermo, Italy; 3 Intensive Care Unit, Department of Medical and Surgical Sciences, University Hospital Mater Domini, Magna Graecia University, Catanzaro, Italy; 4 Value-Based Healthcare Unit, IRCCS MultiMedica, Sesto San Giovanni, Milan, Italy; 5 Emergency Department, “S. Maria Della Misericordia” Hospital, Perugia, Italy; 6 Pulmonology and Respiratory Intensive Care Unit, S. Donato Hospital, Arezzo, Italy; 7 Respiratory Medicine Unit, A.O.U. “Policlinico-Vittorio Emanuele”, Catania, Italy; 8 Pulmonary Rehabilitation Unit, Department of Medicina E Chirurgia, Istituti Clinici Scientifici Maugeri, Università Insubria Varese, Pavia, Italy; 9 Clinical and Experimental Medicine PhD Program, University of Modena and Reggio Emilia, Modena, Italy; 10 Department of Clinical, Integrated and Experimental Medicine (DIMES), Respiratory and Critical Care Unit, S. Orsola-Malpighi Hospital, Alma Mater University, Bologna, Italy; 11 Unit of Emergency Medicine, Department of Medical Sciences, University of Turin, Turin, Italy; 12 Department of Medicine, Clinic of Anesthesia and Intensive Care, University of Udine, Udine, Italy; 13 Respiratory Diseases Unit, Department of Medical and Surgical Sciences SMECHIMAI, University Hospital of Modena Policlinico, University of Modena Reggio Emilia, Modena, Italy; 14 Section of Anesthesiology and Intensive Care, Department of Medicine - DIMED, University of Padova, Padova, Italy; 15 Fondazione ‘Giglio’, Cefalù, Palermo, Italy

**Keywords:** High flow nasal therapy, High flow nasal cannula, Noninvasive ventilation, Chronic obstructive pulmonary disease, Acute respiratory failure

## Abstract

**Background:**

The efficacy and safety of high flow nasal therapy (HFNT) in patients with acute hypercapnic exacerbation of chronic obstructive pulmonary disease (AECOPD) are unclear. Our aim was to evaluate the short-term effect of HFNT versus NIV in patients with mild-to-moderate AECOPD, with the hypothesis that HFNT is non-inferior to NIV on CO_2_ clearance after 2 h of treatment.

**Methods:**

We performed a multicenter, non-inferiority randomized trial comparing HFNT and noninvasive ventilation (NIV) in nine centers in Italy. Patients were eligible if presented with mild-to-moderate AECOPD (arterial pH 7.25–7.35, PaCO_2_ ≥ 55 mmHg before ventilator support). Primary endpoint was the mean difference of PaCO_2_ from baseline to 2 h (non-inferiority margin 10 mmHg) in the per-protocol analysis. Main secondary endpoints were non-inferiority of HFNT to NIV in reducing PaCO_2_ at 6 h in the per-protocol and intention-to-treat analysis and rate of treatment changes.

**Results:**

Seventy-nine patients were analyzed (80 patients randomized). Mean differences for PaCO_2_ reduction from baseline to 2 h were − 6.8 mmHg (± 8.7) in the HFNT and − 9.5 mmHg (± 8.5) in the NIV group (*p* = 0.404). By 6 h, 32% of patients (13 out of 40) in the HFNT group switched to NIV and one to invasive ventilation. HFNT was statistically non-inferior to NIV since the 95% confidence interval (CI) upper boundary of absolute difference in mean PaCO_2_ reduction did not reach the non-inferiority margin of 10 mmHg (absolute difference 2.7 mmHg; 1-sided 95% CI 6.1; *p* = 0.0003). Both treatments had a significant effect on PaCO_2_ reductions over time, and trends were similar between groups. Similar results were found in both per-protocol at 6 h and intention-to-treat analysis.

**Conclusions:**

HFNT was statistically non-inferior to NIV as initial ventilatory support in decreasing PaCO_2_ after 2 h of treatment in patients with mild-to-moderate AECOPD, considering a non-inferiority margin of 10 mmHg. However, 32% of patients receiving HFNT required NIV by 6 h. Further trials with superiority design should evaluate efficacy toward stronger patient-related outcomes and safety of HFNT in AECOPD.

*Trial registration*: The study was prospectively registered on December 12, 2017, in ClinicalTrials.gov (NCT03370666).

## Introduction

Chronic obstructive pulmonary disease (COPD) patients may require respiratory support and hospitalization due to an acute exacerbation of their disease (AECOPD) [1, 2]. To date, noninvasive ventilation (NIV) represents the cornerstone treatment for the management of patients with AECOPD with associated respiratory acidosis [3]. However, several factors may determine NIV failure like discomfort related to the interface, patient–ventilator interaction, airway secretions, the severity of the disease, and the skill of the team of caregivers [4–7].

High flow nasal therapy (HFNT) [8] has been shown to provide potential beneficial effects for patients with stable COPD: It creates a distending pressure generating a positive end-expiratory pressure (PEEP) effect that may counterbalance intrinsic PEEP [9], a washout of nasopharyngeal dead space optimizing ventilatory efficiency and facilitating carbon dioxide removal [10], a reduced inspiratory resistance providing adequate flow and warm gases preventing bronchoconstriction response to dry air [11, 12] enhancing lung mucociliary clearance [13, 14], and finally decreasing diaphragmatic effort in a similar way to NIV [15]. Recent data also showed that HFNT might have a role in managing patients with AECOPD [16]. It has been shown to reduce arterial partial pressure of carbon dioxide (PaCO_2_) [17, 18] and to improve inspiratory effort when used in the NIV interval periods as compared to conventional oxygen therapy [19, 20]. In non-randomized trials, HFNT showed to be equivalent to NIV in avoiding intubation in mild-to-moderate AECOPD patients with respiratory acidosis with similar failure rates, but with better comfort and fewer complications for the patients using HFNT [21, 22]. Thus, there is a reasonable physiological and clinical rationale for using HFNT in AECOPD, but its efficacy and safety are unclear [23].

Our aim was to evaluate the short-term effect of HFNT versus NIV in patients with mild-to-moderate AECOPD, with the hypothesis that HFNT is non-inferior to NIV on CO_2_ clearance after 2 h of treatment.

## Methods

### Study design and patients

This was an investigator-initiated randomized, unblinded, multicenter, non-inferiority, controlled trial conducted from February 15, 2018, to March 25, 2020. The study was conducted in the Emergency Department, Intensive Care Units (ICU), or Respiratory Unit of 9 centers in Italy. The study protocol was approved by “Comitato Etico Sezione Area Centro” Ethics Committee (approval no. 245—October 24, 2017, Catanzaro, Italy) and by the local ethics committee of all the study centers. This study was conducted in accordance with the amended Declaration of Helsinki. Written informed consent was obtained from all patients or legal representatives. The study has been prospectively registered in ClinicalTrials.gov in December 2017 (Identifier: NCT03370666), and the study protocol has been published [24].

We considered eligible adults (i.e., > 18 years/old) patients with a diagnosis of COPD exacerbation according to GOLD criteria [25] admitted for a mild-to-moderate acute hypercapnic respiratory failure, with an arterial pH between 7.25 and 7.35 and a PaCO_2_ ≥ 55 mmHg. Exclusion criteria were: (1) received HFNT or NIV before the study enrolment; (2) long-term domiciliary NIV; (3) clinical cardiovascular instability, as defined by the need for vasopressors, acute coronary syndrome or life-threatening arrhythmias [26]; (4) treatment refusal; 5) agitation, characterized by a Richmond Agitation Sedation Scale (RASS) ≥ 2 or lack of collaboration, defined by a Kelly Matthay score ≥ 5 [26]; (6) acute failure of more than two organs [26]; (7) cardiac arrest; (8) respiratory arrest deeming immediate intubation; (9) recent facial or neck trauma, burns or skin breakdown; (10) pregnancy; (11) consent withdrawal; and (12) enrolment in other research protocols.

### Interventions

In the intervention group, patients received HFNT (Optiflow and MR850 or AIRVO™ 2, Fisher & Paykel Healthcare, Auckland, New Zealand), initially set at 60 L/min, at a temperature of 37 °C. In case of discomfort, flow and/or temperature were down-regulated to the most tolerated setting.

In the control group, patients received NIV through a total full-face or oro-nasal mask. The ventilator was set in Pressure Support Ventilation (PSV) mode, with a PEEP titrated between 3 and 5 cm H_2_O. The inspiratory pressure was titrated to achieve a measured or estimated expiratory tidal volume equal to 6–8 mL kg^−1^ of ideal body weight [26]. The attending physician, based on local availability, selected the ventilators used to deliver NIV.

In both groups, therapeutic management other than ventilatory support was according to current guidelines [25]. Sedation was allowed to improve patients’ comfort and tolerance of the interfaces (target RASS between 0 and -2). The inspired oxygen fraction (FiO_2_) was set to maintain a peripheral oxygen saturation (SpO_2_) target between 88–92% [25]. During study interventions, patients were monitored with continuous SpO_2_, electrocardiogram, and noninvasive blood pressure.

### Study endpoints

The primary endpoint was the mean difference of PaCO_2_ to evaluate the non-inferiority of HFNT to NIV from baseline to 2 h after the randomization. The secondary endpoints were: (1) non-inferiority of HFNT to NIV in reducing PaCO_2_ at 6 h after randomization; (2) treatment change rates (switch to the other study intervention, to IMV, to no support or no change); (3) dyspnea score and proportion of patients who did not improve the dyspnea score; (4) discomfort score and proportion of patients showing poor tolerance to treatment; (5) the proportion of patients who had PaCO_2_ worsening or reduction < 10 mmHg from baseline assessment, or worsening or no improvement of the dyspnea; (6) respiratory rate; (7) change in arterial blood gases; 8) time spent under mechanical ventilation (both IMV and NIV); (9) hospital length of stay; and (10) hospital mortality.

### Data collection and outcome assessment

We collected anthropometric and clinical baseline characteristics, i.e., the Simplified Acute Physiology Score (SAPS II), the Kelly-Matthay Score [27], the Charlson index [28], and the Richmond Agitation Sedation Scale (RASS) [29]. Furthermore, soon before the randomization, we collected the vital parameters, the presence of dyspnea (Borg scale) [30], and the arterial blood gases at patients’ inclusion. All these relevant variables and endpoints were evaluated at 2 and 6 h after randomization. In particular, we recorded: the NIV and HFNT settings; the discomfort related to the interface, as assessed through a ten-point numeric rating scale [31], the proportion of patients reporting poor tolerance to the treatment (defined as a patient-reported complaint to the assigned treatment that did not cause treatment interruption) due to flow, temperature, noise, claustrophobia, sweating, tightness, airway dryness, vomiting gastric distension, ocular irritation, or skin breakdown [31].

The decision to change the assigned treatment was left up to the clinical judgment of the attending physician and was motivated (in unfavorable cases) by any of the following: failure to improve or worsening of clinical signs of respiratory failure or gas exchange (i.e., PaCO_2_ > 20% baseline and/or pH < 7.25) in the case of change from HFNT to NIV; intolerance to the assigned intervention defined as a patient-reported complaint that compromised the pursuit of the treatment (i.e., subject refusal). The decision to intubate was based on the physician’s clinical judgment and on the presence of at least one of the following criteria: respiratory arrest, respiratory apnea or pauses with loss of consciousness, severe agitation, bradycardia (< 50 beats/min) with loss of consciousness, hemodynamic instability with systolic arterial pressure < 70 mmHg, need for invasive mechanical ventilation due to worsening in arterial blood gases and pH decline or pH < 7.25, management of abundant respiratory secretions, intolerance to all the interfaces (including the eventual change of the treatment). Data were collected on a dedicated case report form.

### Randomization and statistical analysis

We computed a sample size of 56 patients, given an alpha error of 5% (one-sided) and a power of 80%, with a standard deviation for the primary outcome equal to 15 mmHg and a non-inferiority limit of 10 mmHg. Thus, the non-inferiority would be demonstrated if the upper boundary of the 95% CI for the mean difference was lower than 10 mmHg. A non-inferiority margin of 10 mmHg was set as a clinically relevant difference in change of PaCO_2_ between the groups by consensus among the investigators, based on clinical judgment and available data at the time of trial design [17, 26, 32, 33]. After considering potential dropouts (30%) and an increase in sample size for nonparametric analysis (15%), the final computed sample size was 80 patients (40 per group). No imputation was planned for missing data.

Randomization was achieved using a computer-generated randomization sequence, generated by an independent investigator, not otherwise involved in the trial, with an allocation ratio of 1:1 and with permuted block method. A single randomization list for all participants was created. Allocation concealment was maintained using sequentially numbered sealed opaque envelopes. Each envelope contained the patient’s allocation to either control (NIV) or intervention (HFNT), with a unique patient identifier code. The randomization was based on a centralized phone call system. Due to the research design, neither the individual collecting data nor the patient can be blinded to treatment allocation. The baseline was defined as the time of randomization.

An independent statistician (FM), blinded to treatment allocation, performed all statistical analyses on per-protocol and intention-to-treat bases as recommended [34]. The per-protocol analysis included only patients who received the randomly assigned intervention till the 2-h (per-protocol analysis at 2 h) and the 6-h assessment (per-protocol analysis at 6 h), excluding those who changed the treatment. The intention-to-treat analysis included all patients according to the randomization, whether they changed the intervention or not at the study timepoints [34].

After checking the skewness of distribution, continuous data will be presented as mean (standard deviation) or median [25th–75th percentile]. Categorical data were expressed as counts and percentages. Differences between treatments in continuous variables were evaluated by the Mann–Whitney *U*-test or the Student *t *test according to Normal distribution. Categorical data were compared with the Chi-square test or Fisher exact test. Paired sample *t *test or paired samples Wilcoxon test were used to assess the difference between timepoints in continuous variables for each group. Change over time was modeled using mixed-effects linear regression with a random intercept and slope (time) in order to account for non-independence among measures. PaCO_2_ values were regressed on time (baseline, 2 h, 6 h), group treatment (HFNT, NIV), and the time per group interaction.

For the primary outcome, a one-sided two-sample Student *t *test was performed while taking into account the non-inferiority margin (10 mmHg) to test whether PaCO_2_ reduction with HFNT was non-inferior to NIV. The same approach was used to evaluate the non-inferiority of HFNT compare to NIV in the PaCO_2_ decrease after 6 h.

A *p* value < 0.05 will be considered significant. Statistical analyses were performed with SAS software, version 9.4 (SAS Institute Inc., Cary, NC, USA) and R, version 3.5.2 (The R Foundation for Statistical Computing) was used for data visualization. Data monitoring was done by two investigators (LB, PC) querying the final database after collecting CRFs from enrolling centers.

## Results

CONSORT checklist for this non-inferiority trial is reported in the Additional file 1.

### Patients and interventions

**Fig. 1:**
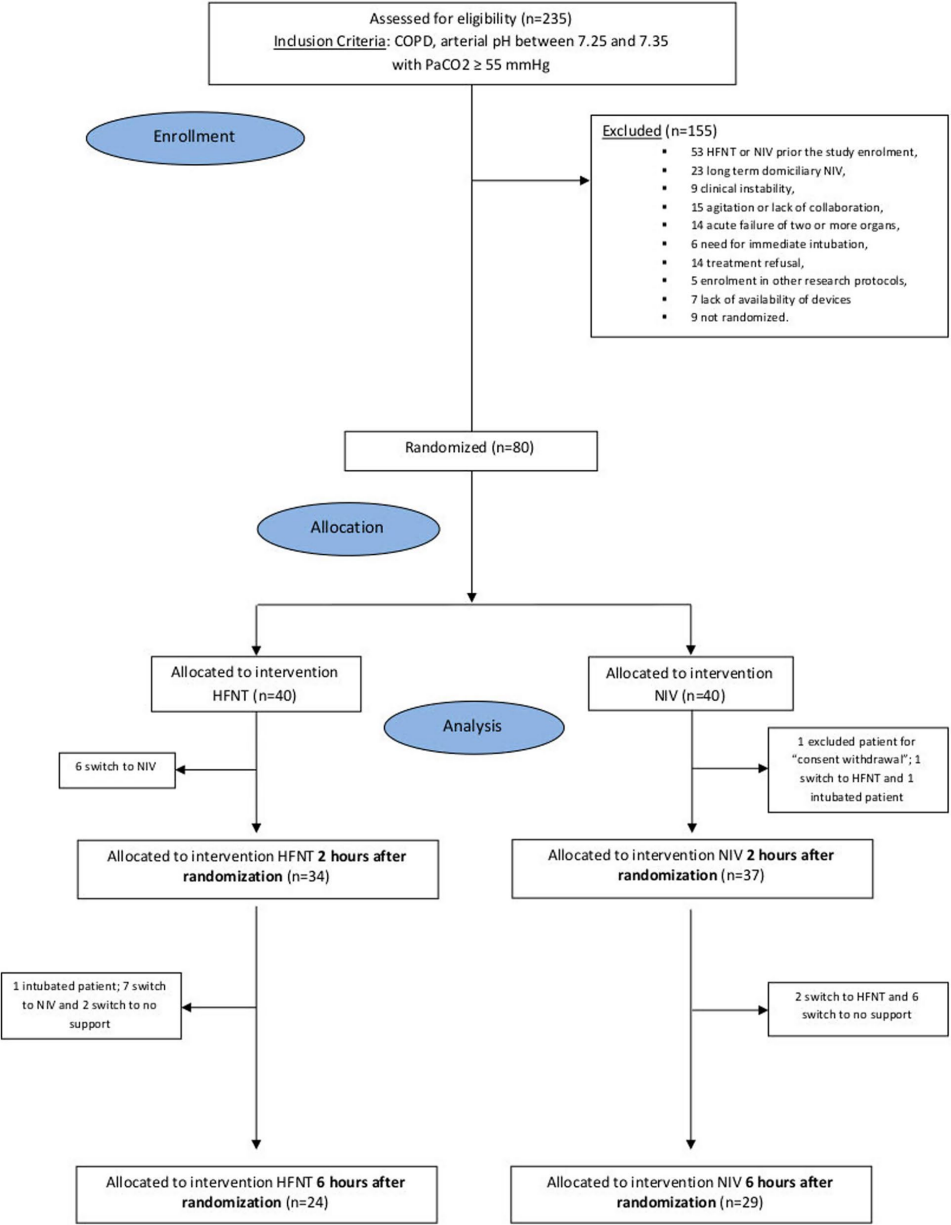
Flow diagram of the trial according to CONSORT

From 235 eligible patients, we randomized 80 patients, 40 in both groups (Fig. 1). The most frequent reason for exclusion that we registered was the prior use of NIV or HFNT before enrollment (53 out of 155 patients excluded). One patient withdrew consent after randomization; therefore, data from 79 patients was analyzed.

Baseline characteristics and blood gases of patients were evenly distributed between the two groups (Table 1). Mean age was 74 (± 13) and 77 (± 12), SAPS II was 30 (± 9.0), and 33 (± 10), the mean arterial pH was 7.30 (± 0.03) and 7.29 (± 0.03), and the mean PaCO_2_ was 73.7 mmHg (± 12.8) and 72.0 mmHg (± 13.0) in the HFNT and NIV group, respectively (Table 1).

**Table 1:** Patients’ characteristics in the high flow nasal therapy (HFNT) and noninvasive ventilation (NIV) groups at baseline

	HFNT group	NIV group	*p *value
*N*	40	39	
Females, *n* (%)	19 (47.5)	20 (51.3)	0.7368
Age (years), mean ± SD	74 ± 13	77. ± 12	0.2273
Weight (kg)^a^, mean ± SD	85 ± 23	76 ± 13	0.0964
Height (m)^a^, mean ± SD	1.7 ± 0.1	1.7 ± 0.1	0.5494
BMI (kg/m^2^)^a^, mean ± SD	30.5 ± 8.7	26.7 ± 5.5	0.0622
Ward of admission, *n* (%)			0.5807
Emergency room	24 (60)	21 (53.8)	
ICU or respiratory unit	16 (40)	18 (46.1)	
SAPS II, mean ± SD	30 ± 9	33 ± 10	0.1497
Charlson index, mean ± SD	4 ± 2	5 ± 3	0.4709
Systolic blood pressure (mmHg), mean ± SD	131 ± 27	137 ± 24	0.3629
Diastolic blood pressure (mmHg), mean ± SD	71 ± 17	70 ± 13	0.7089
Heart rate (per min), mean ± SD	91 ± 20	92 ± 19	0.9609
Respiratory rate (per min), mean ± SD	27 ± 7	28 ± 7	0.5544
Body temperature^b^ (C°), mean ± SD	36.6 ± 0.7	36.8 ± 0.5	0.0489
Kelly Matthay score, *n* (%)			0.0694
Alert, follows complex command (1)	19 (47.5)	25 (64.1)	
Alert, follows simple commands (2)	9 (22.5)	8 (20.5)	
Lethargie (3)	12 (30)	4 (10.3)	
Stuporous (4)	0 (0)	2 (5.1)	
Borg dyspnea score^b^, mean ± SD	5 ± 2	5 ± 2	0.4463
RASS, *n* (%)			0.4813
Light sedation (− 2)	2 (5)	2 (5.1)	
Drowsy (− 1)	13 (32.5)	7 (17.9)	
Alert and calm (0)	21 (52.5)	26 (66.7)	
Restless (+ 1)	4 (10)	4 (10.3)	
Secretion, *n* (%)			0.2163
Normal	23 (57.5)	17 (43.6)	
Abundant	17 (42.5)	22 (56.4)	
PaCO_2_ (mmHg), mean ± SD	73.7 ± 12.8	72.0 ± 13.0	0.5270
Arterial pH, mean ± SD	7.30 ± 0.03	7.29 ± 0.03	0.7450
PaO_2_ (mmHg), mean ± SD	64.3 ± 17.6	73.3 ± 27.9	0.1480
SpO_2_ (%), median [IQR]	90.1 [87.0–94.1]	92.0 [88.0–96.0]	0.1835
HCO_3_^−^ (mmol L^−1^), mean ± SD	34.3 ± 5.9	33.1 ± 6.30	0.3680
PaO_2_/FiO_2_, mean ± SD	203.2 ± 45.5	222.4 ± 71.0	0.4801
Lactate^c^ (mmol L^−1^), median [IQR]	1.1 [0.7–1.6]	1.1 [0.9–1.5]	0.7376

*BMI* body mass index, *FiO*_*2*_ fraction of inspired oxygen, *HCO*_*3*_^*−*^ bicarbonate, *HFNT* high-flow nasal therapy, *ICU* intensive care unit, *IQR* interquartile range (first and third quartile), *NIV* noninvasive ventilation, *PaO*_*2*_arterial partial pressure, *PaCO*_*2*_ partial pressure of carbon dioxide, *RASS* Richmond agitation-sedation scale, *SAPS* simplified acute physiology score, *SD* standard deviation

^a^Data was not available for five patients (three in HFNT and two NIV group)

^b^Data was not available for one patient in NIV group

^c^Data was not available for one patient in HFNT group

All patients received the assigned treatment after randomization. By 2 h, six patients in the HFNT group had switched to NIV (*n* = 5 worsening/no improvement of respiratory failure; *n* = 1 intolerance to the intervention) while one patient switched to HFNT (intolerance to the intervention) and one to IMV in the NIV group (worsening of respiratory failure). By 6 h, seven patients had switched to NIV (worsening/no improvement of respiratory failure), one to IMV (worsening of respiratory failure) in the HFNT group. Due to the improvement of respiratory failure, one patient switched to no support in the HFNT group while two patients switched to HFNT and six to no support in the NIV group.

Seventy-one patients (HFNT *n* = 34 [85%] vs. NIV *n* = 37 [95%]; *p* = 0.2633) and 53 patients (HFNT *n* = 24 [60%] vs. NIV *n* = 29 [74.4%]; *p* = 0.1745) continued the allocated interventions after 2 and 6 h, respectively (Fig. 1, Table 2). Characteristics of interventions are reported in Additional file 2: Table S1. During the 6 h of treatment, three patients in the HFNT (7.5%) and in the NIV (7.7%) received sedatives according to the study protocol.

**Table 2:** Clinical outcomes in the high flow nasal therapy (HFNT) and noninvasive ventilation (NIV) groups

	HFNT group	NIV group	*p *value
*N*	40	39	
Treatment changes from baseline to 2 h, *n* (%)			
Switching to NIV or HFNT	6 (15)	1 (2.6)	0.1084
IMV	0 (0	1 (2.6)	0.4937
No treatment change	34 (85)	37 (94.9)	0.2633
Treatment changes from baseline to 6 h, *n* (%)			
Switch to NIV or HFNT	13 (32.5)	3 (7.7)	0.0061
Switch to IMV	1 (2.5)	1 (2.6)	1.0000
Switch to no support	2 (5)	6 (15.4)	0.1543
No treatment change	24 (60)	29 (74.4)	0.1745
Poor treatment tolerance/intolerance from baseline to 6 hours^a^, *n* (%)			
In patients switching to NIV or HFNT or IMV	5 (35.7)	3 (75)	0.2745
In patients switching to no support or with no treatment change	9 (34.6)	26 (74.3)	0.0019
Discomfort, median [IQR]			
At 2 h^a^	1 [0–2]	3 [1–5]	0.0010
At 6 h^b^	0 [0–2]	2 [1–4]	0.0003
Borg dyspnea score, mean ± SD			
At 2 h^a^	3 ± 2	3 ± 2	0.2509
At 6 h^b^	5 ± 2	5 ± 2	0.4865
No improvement of Borg dyspnea score at 6 h, n (%)	6 (15)	6 (15.4)	0.9620
Respiratory rate (per min), mean ± SD			
At 2 h^b^	22 ± 5	22 ± 4	0.5789
At 6 h^c^	20 ± 4	21 ± 4	0.5573
PaCO_2_ worsening or reduction < 10 mmHg after 6 h, *n* (%)	23 (57.5)	14 (35.9)	0.0544
IMV during hospitalization^d^			
Subjects, *n* (%)	2 (5)	1 (2.6)	1.0000
Length of IMV (hours), median [IQR]	123 [45.5–200]	166 [166–166]	1.0000
NIV during hospitalization			
Subjects, *n* (%)	23 (57.5)	39 (100.0)	< .0001
Length of NIV (hours), median [IQR]	70 [14–142]	48 [18–75]	0.3007
Length of hospital stay (days), median [IQR]			
All patients	10 [9–19]	13 [9–16]	0.6579
Survivors at hospital discharge	10 [9–19]	13 [9–16]	0.5510
Dead at hospital discharge	16 [9–22]	15 [3–19]	0.6150
In-hospital mortality, *n* (%)	2 (5)	6 (15.4)	0.1543

*HFNT* high flow nasal therapy, *IQR* interquartile range [first and third quartile], *IMV* invasive mechanical ventilation, *NIV* noninvasive ventilation

^a^Poor tolerance was defined as patient-reported complaint to the assigned treatment that did not cause treatment interruption. Intolerance was defined as patient-reported complaint that compromised the pursuit of the treatment (i.e., subject refusal)

^b^Outcome evaluated on patients still receiving the assigned treatment at 2 h (34 HFNT, 37 NIV)

^c^Outcome evaluated on patients still receiving the assigned treatment at 6 h (24 HFNT, 29 NIV)

^d^IMV during hospitalization was calculated from baseline from hospital discharge or death

### Per-protocol analysis

**Fig. 2:**
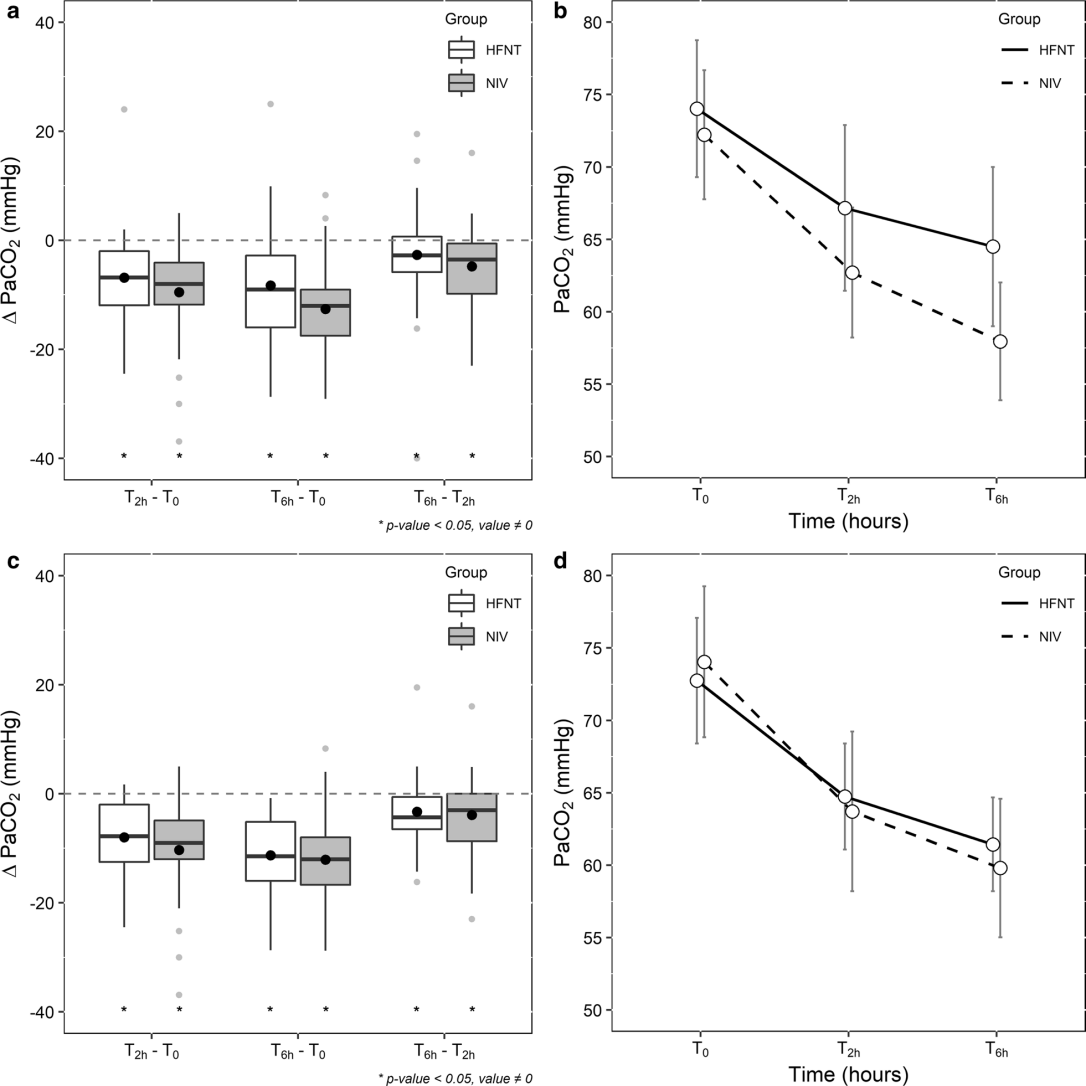
**a**, **c** Report boxplots showing median, interquartile range and mean (full dot) for PaCO_2_ differences (ΔPaCO_2_) between baseline (*T*_0_), 2 h (*T*_2h_) and 6 h (*T*_6h_) in HFNT and NIV groups (*p value < 0.05, difference ≠ 0) in per-protocol analysis at 2 h and at 6 h, respectively; **b**, **d** report mean PaCO_2_ (and 95% confidence interval) observed at *T*_0_, *T*_2h_, *T*_6h_ in HFNT and NIV groups in per-protocol analysis at 2 h and at 6 h, respectively

**Fig. 3:**
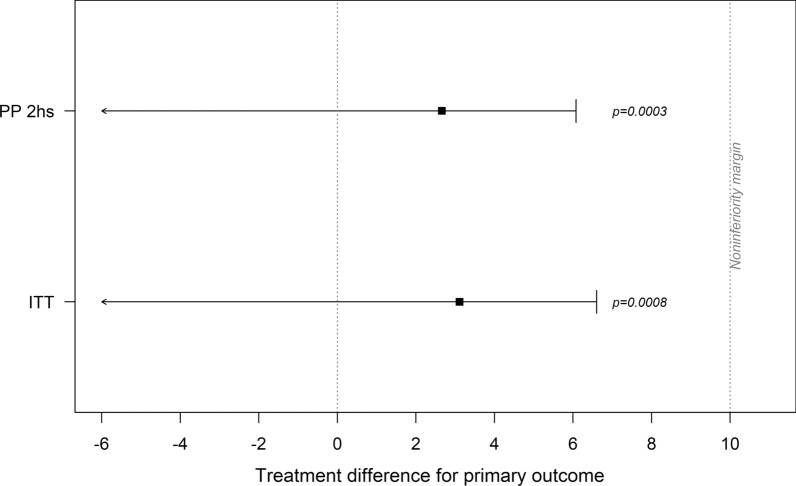
Absolute difference between HFNT and NIV treatment in mean PaCO_2_ reduction after 2 h (and 1-sided 95% confidence interval), according to conducted analyses: per-protocol on patients who completed the treatment originally allocated after 2 h (PP 2 h) and intention-to-treat (ITT). The black box indicates the mean PaCO_2_ reduction after 2 h, full lines indicate 95% confidence interval and dashed line the pre-planned non-inferiority margin of 10 mmHg

At baseline, clinical characteristics and blood gases were similar between the two groups in patients who completed the treatment initially allocated after 2 h (see Additional file 2: Table S2). Mean differences for PaCO_2_ from baseline to 2 h were − 6.8 mmHg (± 8.7) in the HFNT and − 9.5 mmHg (± 8.5) in the NIV group (*p* = 0.404) (Table 3). Figure 2a, b shows the differences and trends in PaCO_2._ Both treatments were able to significantly lower the PaCO_2_ over the study timepoints (Fig. 2a). As regards to the primary outcome, absolute PaCO_2_ difference was 2.7 mmHg (1-sided 95% CI − ∞; 6.1) and HFNT was non-inferior to NIV since the upper boundary of 95% CI did not reach the non-inferiority margin of 10 mmHg (*p* = 0.0003, Fig. 3). Both treatments had a significant effect on PaCO_2_ reduction over time (time effect, *p* < 0.0001), and trends were similar between groups (interaction term, *p* = 0.5864), (Fig. 2b).

**Table 3:** Modifications in PaCO_2_ values during follow-up period in study population stratified by intervention (high flow nasal therapy and noninvasive ventilation)

	HFNT group	NIV group	*p *value
*Per-protocol 2 h*			
PaCO_2_ (mmHg) on patients who completed the treatment originally allocated at 2 h, mean ± SD			
Subjects, *n*	34	37	–
At baseline (* T*_0_)	74.0 ± 13.5	72.2 ± 13.3	0.5845
After 2 h (* T*_2h_ )	67.2 ± 16.4	62.7 ± 13.5	0.1933
After 6 h (* T*_6h_ )	64.5 ± 15.8	57.9 ± 12.2	0.0630
Δ*T*_2h_ − *T*_0_	− 6.8 ± 8.7	− 9.5 ± 8.5	0.4040
Δ*T*_6h_ − *T*_0_	− 9.5 ± 13.0	− 14.3 ± 11.1	0.0962
Δ*T*_6h_ − *T*_2h_	− 2.7 ± 9.7	− 4.8 ± 7.1	0.1637
*Per-protocol 6 h*			
PaCO_2_ (mmHg) on patients who completed the treatment originally allocated at 6 h, mean ± SD			
Subjects, *n*	24	29	−
At baseline (* T*_0_)	72.7 ± 10.3	74.0 ± 13.7	0.7955
After 2 h (* T*_2h_ )	64.7 ± 8.7	63.7 ± 14.5	0.7493
After 6 h (* T*_6h_ )	61.4 ± 7.7	59.8 ± 12.6	0.5632
Δ*T*_2h_ − *T*_0_	− 8.0 ± 6.5	− 10.3 ± 8.9	0.5200
Δ*T*_6h_ − *T*_0_	− 11.3 ± 7.3	− 14.2 ± 12.0	0.4475
Δ*T*_6h_ − *T*_2h_	− 3.3 ± 6.9	− 3.9 ± 7.6	0.8163
*Intention-to-treat analysis*			
PaCO_2_ (mmHg) of enrolled patients, mean ± SD			
Subjects, *n*	40	39	
At baseline (* T*_0_)	73.7 ± 12.8	72.0 ± 13.0	0.5270
After 2 h (* T*_2h_ )	68.2 ± 15.6	63.4 ± 13.6	0.1387
After 6 h (* T*_6h_ )	64.0 ± 14.9	58.1 ± 12.4	0.0610
Δ*T*_2h_ − *T*_0_	− 5.5 ± 9.3	− 8.6 ± 9.3	0.2940
Δ*T*_6h_ − *T*_0_	− 9.7 ± 13.2	− 13.9 ± 11.3	0.1329
Δ*T*_6h_ − *T*_2h_	− 4.2 ± 11.1	− 5.3 ± 7.5	0.3670

*HFNT* high flow nasal therapy, *NIV* noninvasive ventilation, *PaCO*_*2*_ partial pressure of carbon dioxide, *SD* standard deviation, *Δ* difference in PaCO_2_ values between timepoints

Similar results were found in the per-protocol analysis at 6 h (see Fig. 2c, d; Table 3, Additional file 2: Table S3 and Figure S1). However, in this analysis, there was a lower baseline SAPS II score in the HFNT group.

### Intention-to-treat analysis and secondary outcomes

In Additional file 2: Figure S2 (panel A and B) shows the differences and trends in PaCO_2_ for this analysis. Both interventions significantly lower PaCO_2_ over the study timepoints in both groups. Criteria for the non-inferiority of HFNT versus NIV were also met in this analysis at 2 and 6 h (Fig. 3, Additional file 2: Figure S1)

The other secondary outcomes, including dyspnea score, discomfort, length of mechanical ventilation, respiratory rate, the proportion of patients with worsening or no significant reduction in PaCO_2_ values after 6 h, length of hospital stay, and hospital mortality are reported in Table 2.

A higher proportion of patients in the NIV group showed poor tolerance to the intervention by 6 h (74%) compare to HFNT (35%) (*p* = 0.0019). The discomfort score was slightly higher in the NIV group at 2 and 6 h of treatment. For the other secondary outcomes, no differences were found between groups.

Changes in arterial blood gases and vital parameters over the study timepoints for the per-protocol analysis and intention-to-treat analysis were reported in Additional file 2: Tables S4–S6. We found no difference in mean change in arterial pH from baseline to 2 h (HFNT group: 0.04 ± 0.04, NIV group: 0.05 ± 0.03, *p* = 0.39) in the per-protocol analysis at 2 h. In the same analysis, we found a significant difference in mean change of PaO_2_/FiO_2_ ratio (HFNT group: −16.5 ± 52.2 mmHg, NIV group: 7.2 ± 56.3 mmHg, *p* = 0.0357) (see Additional file 2: Table S4).

## Discussion

The main findings of this trial are that HFNT was non-inferior to NIV as initial ventilatory support in mean PaCO_2_ reduction in patients with mild-to-moderate AECOPD considering a non-inferiority margin of 10 mmHg; HFNT was able to reduce PaCO_2_ over both study timepoints significantly, but 32% (14/40) of patients were switched to NIV within 6 h.

To the best of our knowledge, this is the first multicenter randomized controlled non-inferiority trial comparing NIV and HFNT in mild-to-moderate AECOPD. In our study, we included patients with mild-to-moderate AECOPD for two reasons: (1) the efficacy and safety of HFNT in AECOPD have not been demonstrated so far [16]; (2) NIV is considered the gold standard respiratory support for managing patients with AECOPD with high-quality evidence supporting its use [3]. However, HFNT has shown several valuable effects in COPD patients [16] and there are some drawbacks of using NIV, such as reduced comfort and poor patient–ventilator interaction, which is often challenging to recognize and manage [4, 5, 35]. We intended to determine whether HFNT was not inferior to NIV in achieving relevant changes in physiologic outcomes in AECOPD patients (i.e., PaCO_2_) and explore its safety as the first step for future superiority trials.

Previously published studies had shown the promising results on the efficacy and safety of HFNT use for treating AECOPD. A retrospective study performed in a single ICU compared HFNT and NIV in 82 patients with mild-to-moderate AECOPD. No difference was found in the proportion of patients who switched treatment or received IMV (28% HFNT vs. 39% NIV). Of note, patients were assigned to study treatments if they received HFNT or NIV for at least 4 h within the first 24 h from admission [22]. In an observational study performed in one respiratory unit, no difference was found between HFNT and NIV with regard to the 30-day intubation rate (25% vs. 27%) and mortality rate (16% vs. 18%) in 92 patients with moderate AECOPD. The investigators also compared blood gases (including PaCO_2_) at 6- and 24-h, finding no difference between groups [21]. The results included between-group analysis at each timepoint, and no comparison of trends or differences were reported. A randomized study performed in one respiratory unit concluded that HFNT and NIV were both effective in improving blood gases in 168 patients with AECOPD and the HFNT group had a lower rate of complications and higher comfort. No sample size calculation and protocol registration have been reported and the outcomes assessment was done after 12 h and 5 days [36].

We showed that HFNT might be a feasible initial ventilator strategy in the management of mild-to-moderate AECOPD, finding a mean difference in PaCO_2_ reduction of 2.66 mmHg at 2 h, and the 95% CI upper boundary of absolute difference in mean PaCO_2_ reduction not reaching the set non-inferiority margin of 10 mmHg. Nevertheless, we acknowledge that a different non-inferiority margin and a larger sample size would have changed the study conclusions. From a clinical point of view, a clinician may prefer a technique allowing a greater and faster decarboxylation. In fact, 32% of patients treated with HFNT were switched to NIV, even if patients had a slightly lower severity of illness (SAPS II) than those in the NIV group (Table 1 and Additional file 2). Moreover, in our study cohort, patients randomized to HFNT worsened the oxygenation during the 6 h (Additional file 2: Table S4), and those who subsequently underwent NIV during hospitalization had a longer length of NIV than those originally allocated to the NIV group (Table 2). Altogether, these findings should incite caution in declaring that HFNT is non-inferior to NIV from a clinical perspective, all the most because NIV is a true standard of care in this context.

The external validity of our findings is supported by the multicenter design, but the results may have changed according to different expertise in the use of devices, settings and management protocols of AECOPD. Of note, all patients were continuously monitored with standard tools and serial blood gases check, so our findings cannot be extrapolated to settings with different levels of care. Larger clinical trials comparing HFNT and NIV in this patient population are currently ongoing, focusing on other outcomes such as endotracheal intubation (NCT03014869) and treatment failure (NCT03466385) or with crossover design (NCT03033251).

Strengths of the study were the multicenter randomized design, the prospective registration, and protocol publication and the analyses, both per-protocol and intention-to-treat, according to CONSORT recommendations for non-inferiority trials. However, our study has limitations. First, due to the nature of the interventions, blinding was not possible. This study was a non-inferiority trial with a primary physiologic outcome, leaving uncertainty on stronger patient-related outcomes. Moreover, due to the study design, the variables associated with the need for escalation of treatments cannot be adequately evaluated. Although we found lower baseline oxygenation in HFNT patients who escalated the treatment to NIV or IMV after 6 h compared to patients who did not switch (see Additional file 2: Table S7), further studies should assess predictors for HFTN success in this patient population. Our patient cohort was old and the mean BMI in the HFNT group was 30 kg/m^2^ (± 8.7). These factors may limit the external validity of our findings. Furthermore, we cannot exclude the co-presence of obesity-related hypoventilation. It is difficult to screen patients for this condition during the first management AECOPD, but we excluded patients on domiciliary NIV. We did not register the tidal volume generated or estimated by the ventilators in the NIV group. Thus, we cannot exclude that the applied level of pressure support might have affected patients’ comfort and NIV tolerance. Moreover, the PEEP values applied were slightly higher than the range stated in the protocol.

Our study had a parallel-group design with short-term timepoints of assessment, so we cannot evaluate the efficacy and safety of sequential use of the study treatments. The findings that 32% of patients after 6 h and more than 50% of patients during hospitalization needed NIV suggest that the two treatments may have a complementary role in AECOPD that should be further studied [19]. We collected data about the primary outcome, blood gases and secondary outcomes at 6 h as the longest follow-up without further assessments. This can limit the evaluation of the long-term efficacy of HFNT. However, we registered patients’ safety-related outcomes until hospital discharge or death.

At 6 h, the number of patients actually treated as randomly allocated was slightly lower (*n* = 53) than the calculated sample size (*n* = 56) for the primary outcome at 2 h. The power of our per-protocol analysis after 6 h was 76.5%, and therefore, the estimate of the compared effect at this timepoint should be considered with caution. Finally, it has to take into account also the documented clinical heterogeneity of COPD exacerbations [37]; therefore, although we used strict criteria to define mild-to-moderate AECOPD, we may have enrolled patients with different trajectories of the diseases and responses to ventilator support and medical treatments.

## Conclusions

In this trial, HFNT was statistically non-inferior to NIV as initial ventilatory support in decreasing PaCO_2_ after 2 h of treatment in patients with mild-to-moderate AECOPD, considering a non-inferiority margin of 10 mmHg. However, 32% of patients receiving HFNT required NIV by 6 h. Further trials with superiority design should evaluate efficacy toward stronger patient-related outcomes and safety of HFNT in AECOPD.

## Supplementary information

**Additional file 1**: Description of data: CONSORT (consolidated standards of reporting trials) Checklist for Non-inferiority and Equivalence Trials Checklist for Non-inferiority and Equivalence Trials.

** Additional file 2**: Table S1: Characteristics of interventions in the high flow nasal therapy (HFNT) and noninvasive ventilation group (NIV); Table S2: Per-protocol 2 h. Patients’ characteristics in the noninvasive ventilation (NIV) and high flow nasal therapy (HFNT) groups at baseline; Table S3: Per-protocol 6 h. Patients’ characteristics in the noninvasive ventilation (NIV) and high flow nasal therapy (HFNT) groups at baseline; Figure S1: Absolute difference between HFNT and NIV treatment in mean PaCO_2_ reduction after 6 h (and 1-Sided 95% confidence interval), according to conducted analyses: intention-to-treat (ITT) and per-protocol on patients who completed the treatment originally allocated after 6 h (PP 6hs). Figure S2: Changes in PaCO_2_ values during time (intention to treat analysis). Table S4: Per-protocol 2 h. Differences during follow-up in clinical characteristics in the noninvasive ventilation (NIV) and high flow nasal therapy (HFNT) groups; Table S5: Per-protocol 6 h. Differences during follow-up in clinical characteristics in the noninvasive ventilation (NIV) and high flow nasal therapy (HFNT) groups. Table S6: Intention-to-treat analysis. Differences during follow-up in clinical characteristics in the noninvasive ventilation (NIV) and high flow nasal therapy (HFNT) groups. Table S7: reports patients’ characteristics at baseline in the high flow nasal therapy (HFNT) group stratified by success by 6 h.

## Data Availability

The dataset consisting of de-identified participants' data is available from the corresponding author upon reasonable requests.

## Abbreviations

ABGs: Arterial blood gases
AECOPD: Acute exacerbation of chronic obstructive pulmonary disease
CI: Confidence interval
CONSORT: Consolidated standards of reporting trials
COPD: Chronic obstructive pulmonary disease
FiO2: Inspired oxygen fraction
HFNT: High flow nasal therapy
ICU: Intensive care unit
NIV: Noninvasive ventilation
PaCO2: Arterial partial pressure of carbon dioxide
PEEP: Positive end-expiratory pressure
RASS: Richmond Agitation Sedation Scale
SAPS II: Simplified Acute Physiology Score
SpO2: Peripheral oxygen saturation
