# Supplementary material for: High flow nasal therapy versus noninvasive ventilation as initial ventilatory strategy in COPD exacerbation: a multicenter non-inferiority randomized trial
Source: Crit Care. 2020 Dec 14;24:692. doi: 10.1186/s13054-020-03409-0 (PMC7734463; doi:10.1186/s13054-020-03409-0)
Supplement: Supplementary file 2 — Additional file 2: Table S1: Characteristics of interventions in the high flow nasal therapy (HFNT) and noninvasive ventilation group (NIV); Table S2: Per-protocol 2 h. Patients’ characteristics in the noninvasive ventilation (NIV) and high flow nasal therapy (HFNT) groups at baseline; Table S3: Per-protocol 6 h. Patients’ characteristics in the noninvasive ventilation (NIV) and high flow nasal therapy (HFNT) groups at baseline; Figure S1: Absolute difference between HFNT and NIV treatment in mean PaCO2 reduction after 6 h (and 1-Sided 95% confidence interval), according to conducted analyses: intention-to-treat (ITT) and per-protocol on patients who completed the treatment originally allocated after 6 h (PP 6hs). Figure S2: Changes in PaCO2 values during time (intention to treat analysis). Table S4: Per-protocol 2 h. Differences during follow-up in clinical characteristics in the noninvasive ventilation (NIV) and high flow nasal therapy (HFNT) groups; Table S5: Per-protocol 6 h. Differences during follow-up in clinical characteristics in the noninvasive ventilation (NIV) and high flow nasal therapy (HFNT) groups. Table S6: Intention-to-treat analysis. Differences during follow-up in clinical characteristics in the noninvasive ventilation (NIV) and high flow nasal therapy (HFNT) groups. Table S7: reports patients’ characteristics at baseline in the high flow nasal therapy (HFNT) group stratified by success by 6 h. [file 13054_2020_3409_MOESM2_ESM.docx]

**Additional file 2**

**High Flow Nasal Therapy versus Noninvasive Ventilation as initial Ventilatory Strategy in COPD Exacerbation:**

**a Multicenter Non-inferiority Randomized Trial**

Andrea Cortegiani, Federico Longhini, Fabiana Madotto, Paolo Groff, Raffaele Scala, Claudia Crimi, Annalisa Carlucci, Andrea Bruni, Eugenio Garofalo, Santi Maurizio Raineri, Roberto Tonelli, Vittoria Comellini, Enrico Lupia, Luigi Vetrugno, Enrico Clini, Antonino Giarratano, Stefano Nava, Paolo Navalesi, Cesare Gregoretti, and the HF-AECOPD study investigators

**Corresponding author: Corresponding author:** Andrea Cortegiani, MD. Department of Surgical, Oncological and Oral Science (Di.Chir.On.S.), University of Palermo; Department of Anesthesia, Intensive Care and Emergency, Policlinico Paolo Giaccone, University of Palermo, Palermo, Italy. Via del Vespro 129, 90127, Palermo, Italy. Email: andrea.cortegiani@unipa.it ; Phone: +390916552751; Fax: +390916552716

**Table S1:** Characteristics of interventions in the high flow nasal therapy (HFNT) and noninvasive ventilation group (NIV);

**Table S2**: Per-protocol 2 hours. Patients’ characteristics in the noninvasive ventilation (NIV) and high flow nasal therapy (HFNT) groups at baseline;

**Table S3:** Per-protocol 6 hours. Patients’ characteristics in the noninvasive ventilation (NIV) and high flow nasal therapy (HFNT) groups at baseline;

**Figure S1:** Absolute difference between HFNT and NIV treatment in mean PaCO_2_ reduction after 6 hours (and 1-Sided 95% confidence interval), according to conducted analyses: intention-to-treat (ITT) and per-protocol on patients who completed the treatment originally allocated after 6 hours (PP 6hs).

**Figure S2:** Changes in PaCO_2_ values during time (intention to treat analysis).

**Table S4:** Per-protocol 2 hours. Differences during follow-up in clinical characteristics in the noninvasive ventilation (NIV) and high flow nasal therapy (HFNT) groups;

**Table S5:** Per-protocol 6 hours. Differences during follow-up in clinical characteristics in the noninvasive ventilation (NIV) and high flow nasal therapy (HFNT) groups.

**Table S6:** Intention-to-treat analysis. Differences during follow-up in clinical characteristics in the noninvasive ventilation (NIV) and high flow nasal therapy (HFNT) groups.

**Table S7:** reports patients’ characteristics at baseline in the high flow nasal therapy (HFNT) group stratified by success by 6 hours.

**Table S1.** Characteristics of interventions.

| HFNT [n = 40] |  |
| --- | --- |
| Flow (l/min), median [IQR] | 50 [45 – 60] |
| Temperature (C°), median [IQR] | 37 [34 – 37] |
| FiO_2_, median [IQR] | 0.36 [0.30 – 0.44] |
| Cannula size, n (%) |  |
| Small | 4 (10) |
| Medium | 31 (77.5) |
| Large | 5 (12.5) |
| Device, n (%) |  |
| AIRVO | 39 (97.5) |
| OPTIFLOW | 1 (2.5) |
| Brief treatment interruption^a^ | 1 (4) |
| NIV [n = 39] |  |
| Pressure Support (cmH_2_O), median [IQR] | 14 [12 – 16] |
| PEEP (cmH_2_O), median [IQR] | 6 [5 – 6.5] |
| FiO_2_, median [IQR] | 0.30 [0.28 – 0.37] |
| Interface, n (%) |  |
| Oronasal | 24 (61.5) |
| Full-face | 15 (38.5) |
| Brief treatment interruption^b^ | 2 (8) |

^a^The variable is reported as the number of patients who underwent a brief treatment interruption (<10 min.) during the 6 hours. The proportion was calculated on the total who continued HFNT till 6 hours (n=22)

^b^The variable is reported as the number of patients who underwent a brief treatment interruption (< 10 min.) during the 6 hours. The proportion was calculated on the total who continued NIV till 6 hours (n=26)

*Abbreviations: FiO_2_: fraction of inspired oxygen; HFNT: high flow nasal therapy; IQR: interquartile range;*

*NIV: non invasive ventilation; PEEP: positive-end-expiratory pressure.*

**Table S2.** Per-protocol 2 hours. Patients’ characteristics in the noninvasive ventilation (NIV) and high flow nasal therapy (HFNT) groups at baseline.

|  | HFNT group | NIV group | p-value |
| --- | --- | --- | --- |
| N | 34 | 37 |  |
| Females, n (%) | 17 (50) | 19 (51.3) | 0.9094 |
| Age (years), mean ± SD | 74 ± 14 | 77 ± 13 | 0.2134 |
| Weight (kg)^a^, mean ± SD | 86 ± 24 | 76 ± 13 | 0.1113 |
| Height (m)^a^, mean ± SD | 1.7 ± 0.1 | 1.7 ± 0.1 | 0.6288 |
| BMI (kg/m^2^)^a^, mean ± SD | 30.6 ± 9.0 | 27.0 ± 5.6 | 0.1120 |
| Ward of admission, n (%) |  |  | 0.6856 |
| Emergency room | 20 (58.8) | 20 (54) |  |
| ICU or Respiratory Unit | 14 (41.2) | 17 (45.9) |  |
| SAPS II, mean ± SD | 29 ± 8 | 32 ± 10 | 0.0636 |
| Charlson index, mean ± SD | 4 ± 2 | 5 ± 3 | 0.4006 |
| Systolic blood pressure (mmHg), mean ± SD | 135 ± 26 | 137 ± 24 | 0.7351 |
| Diastolic blood pressure (mmHg), mean ± SD | 72 ± 18 | 70 ± 13 | 0.6785 |
| Heart rate (per min), mean ± SD | 92 ± 19 | 92 ± 19 | 0.8223 |
| Respiratory rate (per min), mean ± SD | 27 ± 8 | 28 ± 7 | 0.7469 |
| Body temperature^b^ (C°), mean ± SD | 36.5 ± 0.8 | 36.7 ± 0.5 | 0.0311 |
| Kelly Matthay score, n (%) |  |  | 0.1301 |
| Alert, follows complex command (1) | 17 (50) | 24 (64.9) |  |
| Alert, follows simple commands (2) | 7 (20.6) | 7 (18.9) |  |
| Lethargie (3) | 10 (29.4) | 4 (10.8) |  |
| Stuporous (4) | 0 (0) | 2 (5.4) |  |
| Borg dyspnea score^b^, mean ± SD | 5 ± 2 | 5 ± 2 | 0.5853 |
| RASS, n (%) |  |  | 0.5952 |
| Light sedation (-2) | 2 (5.9) | 2 (5.4) |  |
| Drowsy (-1) | 10 (29.4) | 7 (18.9) |  |
| Alert and calm (0) | 19 (55.9) | 26 (70.3) |  |
| Restless (+1) | 3 (8.8) | 2 (5.4) |  |
| Secretion, n (%) |  |  | 0.4028 |
| Normal | 19 (55.9) | 17 (45.9) |  |
| Abnormal | 15 (44.1) | 20 (54) |  |
| PaCO_2_ (mmHg), mean ± SD | 74.0 ± 13.5 | 72.2 ± 13.3 | 0.5845 |
| Arterial pH, mean ± SD | 7.30 ± 0.03 | 7.29 ± 0.03 | 0.9170 |
| PaO_2_ (mmHg), mean ± SD | 66.7 ± 17.6 | 72.9 ± 26.0 | 0.3662 |
| SpO_2_ (%), median [IQR] | 90.1 ± 7.7 | 90.7 ± 8.5 | 0.5644 |
| HCO_3_^-^ (mmol·L^-1^), mean ± SD | 34.2 ± 6.3 | 33.1 ± 6.4 | 0.4915 |
| PaO_2_/FiO_2_, mean ± SD | 207.4 ± 44.8 | 222.4 ± 70.7 | 0.6702 |
| Lactate^c^ (mmol·L^-1^), median [IQR] | 1.0 [0.7-1.5] | 1.11 [1-1.5] | 0.1931 |

*Abbreviations. BMI: body mass index; FiO_2_: fraction of inspired oxygen; HCO_3_^-^: bicarbonate; HFNT: high flow nasal therapy; ICU: intensive care unit; IQR: interquartile range [first and third quartile]; NIV: non invasive ventilation; PaO_2_: arterial partial pressure; PaCO_2_: partial pressure of carbon dioxide; RASS: Richmond agitation-sedation scale; SAPS: simplified acute physiology score; SD: standard deviation*

*^a^ Data was not available for 3 patients (1 in HFNT and 2 NIV group).^b^ Data was not available for 1 patient in NIV group. ^c^ Data was not available for 1 patient in HFNT group.*

**Table S3.** Per-protocol 6 hours. Patients’ characteristics in the noninvasive ventilation (NIV) and high flow nasal therapy (HFNT) groups at baseline.

|  | HFNT group | NIV group | p-value |
| --- | --- | --- | --- |
| N | 24 | 29 |  |
| Females, n (%) | 10 (41.7) | 17 (58.6) | 0.2191 |
| Age (years), mean ± SD | 74 ± 13 | 79 ± 12 | 0.1114 |
| Weight (kg)^a^, mean ± SD | 84 ± 230 | 76 ± 13 | 0.3189 |
| Height (m)^a^, mean ± SD | 1.7 ± 0.1 | 1.7 ± 0.1 | 0.9848 |
| BMI (kg/m^2^)^a^, mean ± SD | 29.6 ± 8.3 | 27.4 ± 5.8 | 0.5137 |
| Ward of admission, n (%) |  |  | 0.8593 |
| Emergency room | 13 (54.2) | 15 (51.7) |  |
| ICU or Respiratory Unit | 11 (45.8) | 14 (48.3) |  |
| SAPS II, mean ± SD | 27 ± 9 | 33 ± 9 | 0.0159 |
| Charlson index, mean ± SD | 5 ± 2 | 6 ± 3 | 0.1545 |
| Systolic blood pressure (mmHg), mean ± SD | 135 ± 23 | 138 ± 25 | 0.6037 |
| Diastolic blood pressure (mmHg), mean ± SD | 72 ± 15 | 70 ± 14 | 0.5441 |
| Heart rate (per min), mean ± SD | 93 ± 18 | 94 ± 20 | 0.8154 |
| Respiratory rate (per min), mean ± SD | 26 ± 8 | 28 ± 7 | 0.5272 |
| Body temperature^b^ (C°), mean ± SD | 36.4 ± 0.7 | 36.7 ± 0.4 | 0.0257 |
| Kelly Matthay score, n (%) |  |  | 0.3873 |
| Alert, follows complex command (1) | 13 (54.2) | 18 (62.1) |  |
| Alert, follows simple commands (2) | 5 (20.8) | 6 (20.7) |  |
| Lethargie (3) | 6 (25) | 3 (10.3) |  |
| Stuporous (4) | 0 (0) | 2 (6.9) |  |
| Borg dyspnea score^b^, mean ± SD | 5 ± 2 | 5 ± 2 | 0.4865 |
| RASS, n (%) |  |  | 0.4423 |
| Light sedation (-2) | 0 (0) | 2 (6.9) |  |
| Drowsy (-1) | 8 (33.3) | 5 (17.2) |  |
| Alert and calm (0) | 15 (62.5) | 20 (69) |  |
| Restless (+1) | 1 (4.2) | 2 (6.9) |  |
| Secretion, n (%) |  |  | 0.9005 |
| Normal | 12 (50) | 14 (48.3) |  |
| Abnormal | 12 (50) | 15 (51.7) |  |
| PaCO_2_ (mmHg), mean ± SD | 72.7 ± 10.3 | 74.0 ± 13.7 | 0.7955 |
| Arterial pH, mean ± SD | 7.31 ± 0.03 | 7.29 ± 0.03 | 0.1900 |
| PaO_2_ (mmHg), mean ± SD | 69.1 ± 18.8 | 74.9 ± 27.0 | 0.5614 |
| SpO_2_ (%), median [IQR] | 92.7 [90.0-96.0] | 92.0 [89.0-96.0] | 0.9500 |
| HCO_3_^-^ (mmol·L^-1^), mean ± SD | 34.5 ± 5.7 | 34.0 ± 6.6 | 0.7488 |
| PaO_2_/FiO_2_, mean ± SD | 210.7 ± 51.7 | 223.9 ± 76.6 | 0.4757 |
| Lactate (mmol·L^-1^), median [IQR] | 1.0 [0.7-1.3] | 1.1 [0.9-1.4] | 0.2953 |

*Abbreviations. BMI: body mass index; FiO_2_: fraction of inspired oxygen; HCO_3_^-^: bicarbonate; HFNT: high flow nasal therapy; ICU: intensive care unit; IQR: interquartile range [first and third quartile]; NIV: non invasive ventilation; PaO_2_: arterial partial pressure; PaCO_2_: partial pressure of carbon dioxide; RASS: Richmond agitation-sedation scale; SAPS: simplified acute physiology score; SD: standard deviation*

*^a^ Data was not available for 2 patients (1 in HFNT and 1 NIV group). ^b^ Data was not available for 1 patient in NIV group.*

**Figure S1:** Absolute difference between HFNT and NIV treatment in mean PaCO_2_ reduction after 6 hours (and 1-Sided 95% confidence interval), according to conducted analyses: intention-to-treat (ITT) and per-protocol on patients who completed the treatment originally allocated after 6 hours (PP 6hs). The black box indicates the mean PaCO_2_ reduction and the right end of the line indicated the upper bound of the 95% CI. The figure also showed the pre-planned non-inferiority margin (10 mmHg, dashed line).

*Note: For PP 6hs, power is 76.2%.***
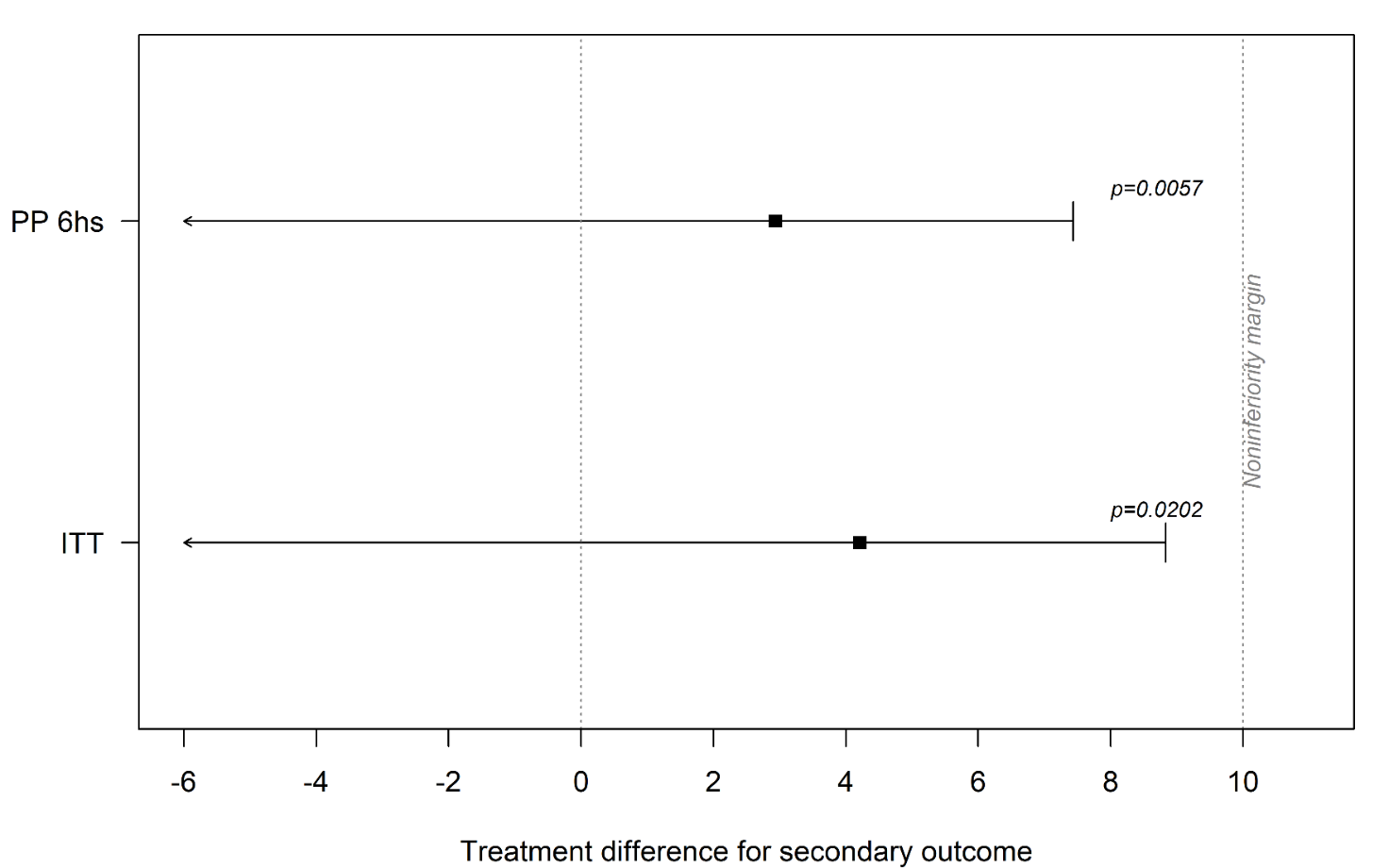
**

**Figure S2:** Changes in PaCO_2_ values during time (intention to treat analysis).

**Panel A.** Boxplot of PaCO_2_ differences in NIV and HFNV patients.

**Panel B.** Trend in mean PaCO_2_ during time in NIV and HFNV patients.

**
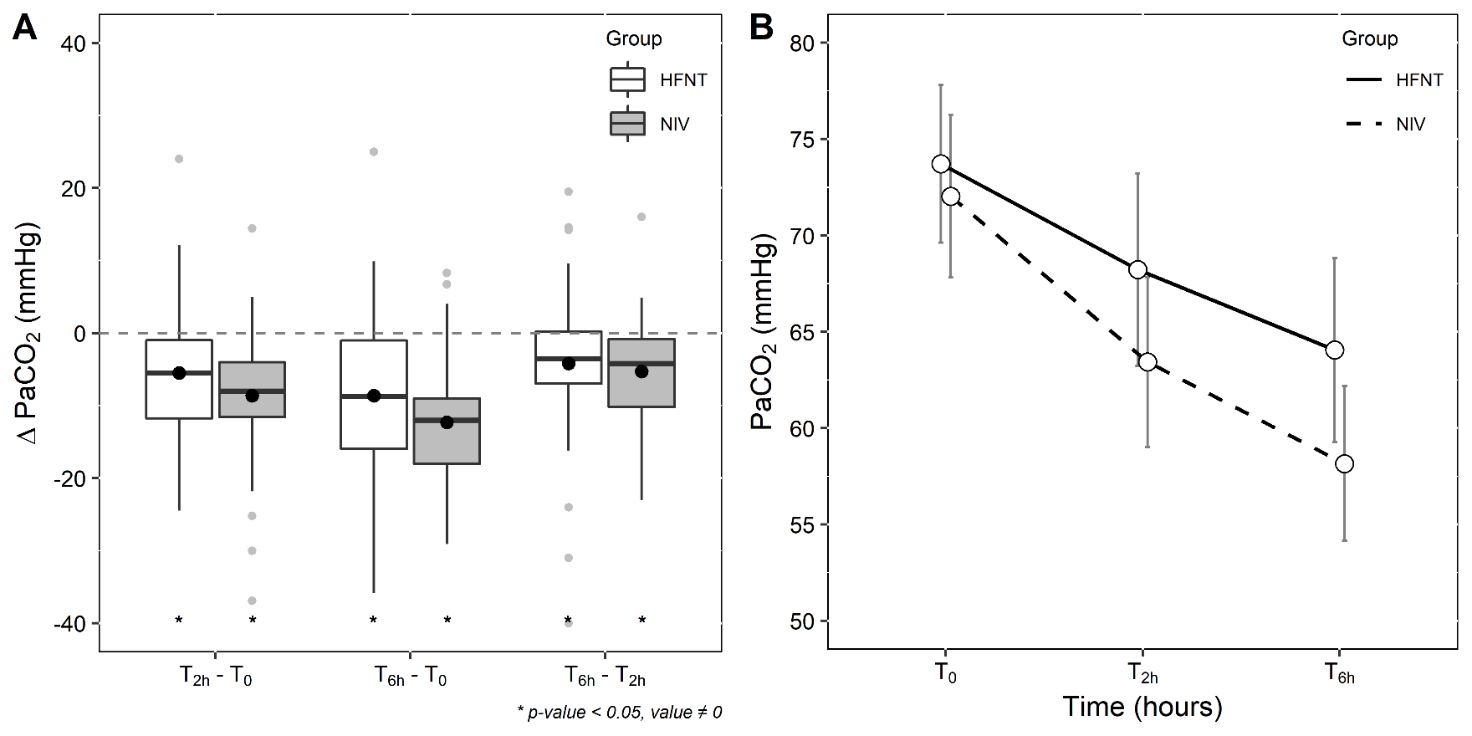
**

**Table S4.** Per-protocol 2 hours. Differences during follow-up in clinical characteristics in the noninvasive ventilation (NIV) and high flow nasal therapy (HFNT) groups.

|  | HFNT group | NIV group | p-value |
| --- | --- | --- | --- |
| N | 34 | 37 |  |
| Systolic blood pressure (mmHg), mean ± SD |  |  |  |
| Δ 2h – baseline | -6.8 ± 24.9 | -10.3 ± 22.8 | 0.8857 |
| Δ 6h – baseline | -9.3 ± 24.1 | -10.0 ± 21.8 | 0.8309 |
| Δ 6h – 2h | -2.5 ± 21.1 | 0.2 ± 14.1 | 0.2437 |
| Diastolic blood pressure (mmHg), mean ± SD |  |  |  |
| Δ 2h – baseline | -1.7 ± 12.2 | -1.3 ± 13.3 | 0.9073 |
| Δ 6h – baseline | 2.9 ± 22.9 | 1.3 ± 11.0 | 0.9076 |
| Δ 6h – 2h | 4.6 ± 21.2 | 2.6 ± 9.5 | 0.7503 |
| Heart rate (per min), mean ± SD |  |  |  |
| Δ 2h – baseline | -5.9 ± 12.7 | -5.8 ± 10.0 | 0.9790 |
| Δ 6h – baseline | -6.5 ± 13.7 | -8.2 ± 13.5 | 0.4787 |
| Δ 6h – 2h | -0.6 ± 10.6 | -2.4 ± 9.6 | 0.2968 |
| Respiratory rate (per min), mean ± SD |  |  |  |
| Δ 2h – baseline | -5.3 ± 4.8 | -5.5 ± 5.8 | 0.8655 |
| Δ 6h – baseline | -5.8 ± 6.2 | -7.4 ± 6.8 | 0.3011 |
| Δ 6h – 2h | -0.4 ± 4.2 | -1.8 ± 3.3 | 0.2903 |
| Arterial pH, mean ± SD |  |  |  |
| Δ 2h – baseline | 0.04 ± 0.04 | 0.05 ± 0.03 | 0.3900 |
| Δ 6h – baseline | 0.06 ± 0.06 | 0.08 ± 0.06 | 0.1151 |
| Δ 6h – 2h | 0.02 ± 0.05 | 0.03 ± 0.04 | 0.2254 |
| PaO_2_ (mmHg), mean ± SD |  |  |  |
| Δ 2h – baseline | 0.4 ± 20.0 | -1.4 ± 29.4 | 0.7255 |
| Δ 6h – baseline | 0.7 ± 20.6 | -2.9 ± 27.1 | 0.4040 |
| Δ 6h – 2h | 0.2 ± 13.4 | -1.5 ± 16.8 | 0.7560 |
| SpO_2_ (%), median [IQR] |  |  |  |
| Δ 2h – baseline | 0.0 [1.0-4.0] | -0.2 [-2.0-5.0] | 0.7515 |
| Δ 6h – baseline | 2.0 [-1.0-5.4] | 1.2 [-2.2-5.0] | 0.5531 |
| Δ 6h – 2h | 1.1 [-1.0-3.0] | 0.3 [-1.2-3.0] | 0.4448 |
| HCO3^-^ (mmol·L^-1^), mean ± SD |  |  |  |
| Δ 2h – baseline | -0.8 ± 4.0 | -0.7 ± 3.5 | 0.8629 |
| Δ 6h – baseline | -0.8 ± 4.2 | -1.1 ± 4.9 | 0.8539 |
| Δ 6h – 2h | 0.02 ± 3.5 | -0.4 ± 3.3 | 0.9358 |
| PaO_2_/FiO_2_, mean ± SD |  |  |  |
| Δ 2h – baseline | -16.5 ± 52.2 | 7.2 ± 56.3 | 0.0357 |
| Δ 6h – baseline | -10.0 ± 61.1 | 17.3 ± 71.4 | 0.0891 |
| Δ 6h – 2h | 6.5 ± 61.8 | 10.1 ± 57.4 | 0.6828 |
| Lactate (mmol·L^-1^), median [IQR] |  |  |  |
| Δ 2h – baseline^a^ | -0.1 [-0.3-0.1] | -0.10 [-0.2-0.1] | 0.6848 |
| Δ 6h – baseline^a^ | 0.0 [-0.4-0.2] | -0.21 [-0.4-0.1] | 0.4642 |
| Δ 6h – 2h^b^ | 0.0 [-0.3-0.2] | 0.00 [-0.2-0.2] | 0.9849 |

*Abbreviations. FiO_2_: fraction of inspired oxygen; HCO_3_^-^: bicarbonate; HFNT: high flow nasal therapy; IQR: interquartile range [first and third quartile]; NIV: non invasive ventilation; PaO_2_: arterial partial pressure; PaCO_2_: partial pressure of carbon dioxide; RASS: Richmond agitation-sedation scale; SD: standard deviation; Δ: difference in values between time points.*

*^a^ Data was not available for 3 patients (2 in HFNT and 1 NIV group).* *^b^ Data was not available for 4 patients (2 in HFNT and 2 NIV group).*

**Table S5.** Per-protocol 6 hours. Differences during follow-up in clinical characteristics in the noninvasive ventilation (NIV) and high flow nasal therapy (HFNT) groups.

|  | HFNT group | NIV group | p-value |
| --- | --- | --- | --- |
| N | 24 | 29 |  |
| Systolic blood pressure (mmHg), mean ± SD |  |  |  |
| Δ 2h – baseline | -7.2 ± 23.2 | -10.2 ± 23.2 | 0.9286 |
| Δ 6h – baseline | -7.0 ± 19.4 | -10.69 ± 24.1 | 0.8158 |
| Δ 6h – 2h | 0.3 ± 22.7 | -0.52 ± 11.7 | 0.6803 |
| Diastolic blood pressure (mmHg), mean ± SD |  |  |  |
| Δ 2h – baseline | -2.0 ± 11.3 | -0.9 ± 14.2 | 0.7201 |
| Δ 6h – baseline | 4.9 ± 24.9 | 1.1 ± 12.0 | 0.5471 |
| Δ 6h – 2h | 6.9 ± 24.2 | 2.1 ± 9.8 | 0.6214 |
| Heart rate (per min), mean ± SD |  |  |  |
| Δ 2h – baseline | -7.5 ± 13.8 | -6.3 ± 10.8 | 0.7164 |
| Δ 6h – baseline | -7.0 ± 13.9 | -8.3 ± 14.0 | 0.7203 |
| Δ 6h – 2h | 0.6 ± 9.5 | -2.0 ± 10.5 | 0.3498 |
| Respiratory rate (per min), mean ± SD |  |  |  |
| Δ 2h – baseline | -5.6 ± 4.6 | -5.7 ± 5.0 | 0.9159 |
| Δ 6h – baseline | -6.1 ± 5.9 | -6.9 ± 6.6 | 0.6601 |
| Δ 6h – 2h | -0.5 ± 4.2 | -1.2 ± 3.3 | 0.9710 |
| Arterial pH, mean ± SD |  |  |  |
| Δ 2h – baseline | 0.04 ± 0.03 | 0.05 ± 0.03 | 0.6286 |
| Δ 6h – baseline | 0.07 ± 0.05 | 0.07 ± 0.05 | 0.8651 |
| Δ 6h – 2h | 0.02 ± 0.03 | 0.02 ± 0.04 | 0.9565 |
| PaO_2_ (mmHg), mean ± SD |  |  |  |
| Δ 2h – baseline | -2.4 ± 18.3 | -1.8 ± 32.2 | 0.9786 |
| Δ 6h – baseline | -0.7 ± 20.5 | -3.5 ± 29.6 | 0.6422 |
| Δ 6h – 2h | 1.8 ± 11.3 | -1.7 ± 17.0 | 0.6810 |
| SpO_2_ (%), median [IQR] |  |  |  |
| Δ 2h – baseline | -0.1 [-1.5-2.6] | -0.2 [-2.0-4.6] | 0.9501 |
| Δ 6h – baseline | 1.2 [-1.9-4.6] | 1.2 [-3.7-5.0] | 0.9929 |
| Δ 6h – 2h | 1.2 [-1.0-3.2] | 0.0 [-1.9-2.0] | 0.4330 |
| HCO3^-^ (mmol·L^-1^), mean ± SD |  |  |  |
| Δ 2h – baseline | -0.5 ± 3.5 | -1.3 ± 3.6 | 0.4110 |
| Δ 6h – baseline | -0.4 ± 3.1 | -1.7 ± 5.1 | 0.3960 |
| Δ 6h – 2h | 0.1 ± 2.2 | -0.4 ± 3.1 | 0.4552 |
| PaO_2_/FiO_2_, mean ± SD |  |  |  |
| Δ 2h – baseline | -12.1 ± 47.3 | 9.0 ± 61.4 | 0.1744 |
| Δ 6h – baseline | 3.3 ± 60.5 | 18.2 ± 73.1 | 0.4286 |
| Δ 6h – 2h | 15.4 ± 58.4 | 9.2 ± 56.9 | 0.3574 |
| Lactate (mmol·L^-1^), median [IQR] |  |  |  |
| Δ 2h – baseline^a^ | -0.1 [-0.2-0.1] | 0.0 [-0.2-0.1] | 0.9773 |
| Δ 6h – baseline^b^ | 0.0 [-0.4-0.1] | -0.1 [-0.4-0.1] | 0.8976 |
| Δ 6h – 2h^c^ | 0.0 [-0.2-0.2] | 0.0 [-0.3-0.3] | 0.7110 |

*Abbreviations. FiO_2_: fraction of inspired oxygen; HCO_3_^-^: bicarbonate; HFNT: high flow nasal therapy; IQR: interquartile range [first and third quartile]; NIV: non invasive ventilation; PaO_2_: arterial partial pressure; PaCO_2_: partial pressure of carbon dioxide; RASS: Richmond agitation-sedation scale; SD: standard deviation; Δ: difference in values between time points.*

*^a^ Data was not available for 2 patients (1 in HFNT and 1 NIV group). ^b^ Data was not available for 1 patient (NIV group).* *^c^ Data was not available for 3 patients (1 in HFNT and 2 NIV group).*

**Table S6.** Intention-to-treat analysis. Differences during follow-up in clinical characteristics in the noninvasive ventilation (NIV) and high flow nasal therapy (HFNT) groups.

|  | HFNT group | NIV group | p-value |
| --- | --- | --- | --- |
| N | 40 | 39 |  |
| Systolic blood pressure (mmHg), mean ± SD |  |  |  |
| Δ 2h – baseline | -5.2 ± 24.3 | -10.0 ± 22.3 | 0.5260 |
| Δ 6h – baseline | -7.6 ± 23.0 | -10.4 ± 21.3 | 0.7015 |
| Δ 6h – 2h | -2.4 ± 20.4 | -0.4 ± 13.9 | 0.3554 |
| Diastolic blood pressure (mmHg), mean ± SD |  |  |  |
| Δ 2h – baseline | -1.5 ± 11.6 | -1.9 ± 13.9 | 0.8941 |
| Δ 6h – baseline | 2.2 ± 21.5 | 0.6 ± 11.2 | 0.8436 |
| Δ 6h – 2h | 3.7 ± 20.0 | 2.6 ± 9.3 | 0.6537 |
| Heart rate (per min), mean ± SD |  |  |  |
| Δ 2h – baseline | -6.1 ± 15.6 | -5.3 ± 10.1 | 0.9922 |
| Δ 6h – baseline | -6.1 ± 16.5 | -7.7 ± 13.4 | 0.6344 |
| Δ 6h – 2h | 0.0 ± 10.6 | -2.4 ± 9.4 | 0.1661 |
| Respiratory rate (per min), mean ± SD |  |  |  |
| Δ 2h – baseline | -4.9 ± 5.0 | -5.6 ± 5.6 | 0.5818 |
| Δ 6h – baseline | -6.0 ± 6.0 | -7.7 ± 6.9 | 0.2558 |
| Δ 6h – 2h | -1.1 ± 4.7 | -2.1 ± 3.7 | 0.5300 |
| Arterial pH, mean ± SD |  |  |  |
| Δ 2h – baseline | 0.03 ± 0.04 | 0.04 ± 0.04 | 0.2564 |
| Δ 6h – baseline | 0.06 ± 0.06 | 0.08 ± 0.06 | 0.1812 |
| Δ 6h – 2h | 0.03 ± 0.06 | 0.03 ± 0.04 | 0.6506 |
| PaO_2_ (mmHg), mean ± SD |  |  |  |
| Δ 2h – baseline | 4.0 ± 21.8 | -0.3 ± 29.1 | 0.4741 |
| Δ 6h – baseline | 3.1 ± 20.7 | -2.6 ± 26.6 | 0.1760 |
| Δ 6h – 2h | -0.9 ± 14.8 | -2.3 ± 16.7 | 0.7058 |
| SpO_2_ (%), median [IQR] |  |  |  |
| Δ 2h – baseline | 0.4 [-0.8-5.5] | -0.2 [-2.0-5.0] | 0.3290 |
| Δ 6h – baseline | 3.0 [-1.0-6.0] | 1.0 [-2.2-5.0] | 0.1479 |
| Δ 6h – 2h | 1.1 [-1.1-3.2] | 0.3 [-1.9-3.0] | 0.3554 |
| HCO3^-^ (mmol·L^-1^), mean ± SD |  |  |  |
| Δ 2h – baseline | -0.9 ± 3.8 | -0.8 ± 3.5 | 0.7874 |
| Δ 6h – baseline | -0.7 ± 4.1 | -1.2 ± 4.8 | 0.6239 |
| Δ 6h – 2h | 0.1 ± 3.5 | -0.4 ± 3.2 | 0.7314 |
| PaO_2_/FiO_2_, mean ± SD |  |  |  |
| Δ 2h – baseline | -17.2 ± 52.7 | 6.8 ± 54.9 | 0.0504 |
| Δ 6h – baseline | -2.2 ± 62.3 | 17.8 ± 70.8 | 0.1826 |
| Δ 6h – 2h | 15.0 ± 62.7 | 11.0 ± 58.0 | 0.3541 |
| Lactate (mmol·L^-1^), median [IQR] |  |  |  |
| Δ 2h – baseline^a^ | -0.1 [-0.4-0.1] | -0.1 [-0.2-0.03] | 0.8353 |
| Δ 6h – baseline^a^ | 0.0 [-0.5-0.2] | -0.2 [-0.4-0.1] | 0.8191 |
| Δ 6h – 2h^b^ | 0.0 [-0.3-0.2] | 0.0 [-0.22-0.2] | 0.8987 |

*Abbreviations. FiO_2_: fraction of inspired oxygen; HCO_3_^-^: bicarbonate; HFNT: high flow nasal therapy; IQR: interquartile range [first and third quartile]; NIV: non invasive ventilation; PaO_2_: arterial partial pressure; PaCO_2_: partial pressure of carbon dioxide; RASS: Richmond agitation-sedation scale; SD: standard deviation; Δ: difference in values between time points.*

*^a^ Data was not available for 3 patients (2 in HFNT and 1 NIV group). ^b^ Data was not available for 4 patients (2 in HFNT and 2 NIV group).*

**Table S7.** Patients’ characteristics at baseline in the high flow nasal therapy (HFNT) group stratified by success by 6 hours.

|  | No switch or switch to no support | Switch to IMV or NIV | p-value |
| --- | --- | --- | --- |
| N | 26 | 14 |  |
| Females, n (%) | 11 (42.3) | 8 (57.1) | 0.3702 |
| Age (years), mean ± SD | 74 ± 12 | 73.5 ± 15 | 0.8425 |
| Weight (kg)^a^, mean ± SD | 82 ± 23 | 91 ± 24 | 0.2227 |
| Height (m)^a^, mean ± SD | 1.7 ± 0.1 | 1.7 ± 0.1 | 0.6592 |
| BMI (kg/m^2^)^a^, mean ± SD | 29.2 ± 8.3 | 33.1 ± 9.3 | 0.1442 |
| Ward of admission, n (%) |  |  | 0.2790 |
| Emergency room | 14 (53.8) | 10 (71.4) |  |
| ICU or Respiratory Unit | 12 (46.1) | 4 (28.6) |  |
| SAPS II, mean ± SD | 28 ± 9 | 33 ± 9 | 0.1256 |
| Charlson index, mean ± SD | 4 ± 2 | 4 ± 2 | 0.6990 |
| Systolic blood pressure (mmHg), mean ± SD | 138 ± 25 | 119 ± 26 | 0.0379 |
| Diastolic blood pressure (mmHg), mean ± SD | 74 ± 17 | 15 ± 0.2 | 0.1406 |
| Heart rate (per min), mean ± SD | 94 ± 19 | 87 ± 24 | 0.3312 |
| Respiratory rate (per min), mean ± SD | 27 ± 8 | 27. ± 6 | 0.7894 |
| Body temperature (C°), mean ± SD | 36.5 ± 0.8 | 36.6 ± 0.6 | 0.4322 |
| Kelly Matthay score, n (%) |  |  | 0.4687 |
| Alert, follows complex command (1) | 14 (53.8) | 5 (35.7) |  |
| Alert, follows simple commands (2) | 6 (23.1) | 3 (21.4) |  |
| Lethargie (3) | 6 (23.1) | 6 (42.9) |  |
| Borg dyspnea score, mean ± SD | 5 ± 2 | 5 ± 2 | 0.9543 |
| RASS, n (%) |  |  | 0.0242 |
| Light sedation (-2) | 0 (0) | 2 (14.3) |  |
| Drowsy (-1) | 8 (30.8) | 5 (35.7) |  |
| Alert and calm (0) | 17 (65.4) | 4 (28.6) |  |
| Restless (+1) | 1 (3.8) | 3 (21.4) |  |
| Secretion, n (%) |  |  | 0.5241 |
| Normal | 14 (53.8) | 9 (64.3) |  |
| Abnormal | 12 (46.1) | 5 (35.7) |  |
| PaCO_2_ (mmHg), mean ± SD | 71.7 ± 10.5 | 77.4 ± 16.0 | 0.4438 |
| Arterial pH, mean ± SD | 7.30 ± 0.03 | 7.28 ± 0.04 | 0.0540 |
| PaO_2_ (mmHg), mean ± SD | 68.8 ± 18.1 | 56.0 ± 13.8 | 0.0040 |
| SpO_2_ (%), median [IQR] | 92.1 [90.0-96.0] | 87.0 [82.9-88.0] | 0.0009 |
| HCO_3_^-^ (mmol·L^-1^), mean ± SD | 33.9 ± 5.9 | 35.0 ± 5.9 | 0.5873 |
| PaO_2_/FiO_2_, mean ± SD | 211.5 ± 49.8 | 187.9 ± 32.7 | 0.1193 |
| Lactate^b^ (mmol·L^-1^), median [IQR] | 1.1 [0.7-1.3] | 1.5 [0.9-2.0] | 0.2443 |

*Abbreviations. BMI: body mass index; FiO_2_: fraction of inspired oxygen; HCO_3_^-^: bicarbonate; HFNT: high flow nasal therapy; ICU: intensive care unit; IQR: interquartile range [first and third quartile]; NIV: non invasive ventilation; PaO_2_: arterial partial pressure; PaCO_2_: partial pressure of carbon dioxide; RASS: Richmond agitation-sedation scale; SAPS: simplified acute physiology score; SD: standard deviation*

*^a^ Data was not available for 3 patients (1 in “No switch or switch to no support” and 2 “Switch to IMV or NIV” group). ^b^ Data was not available for 1 patient in “Switch to IMV or NIV” group.*
